# Tongue squamous cell carcinoma producing both parathyroid hormone-related protein and granulocyte colony-stimulating factor: a case report and literature review

**DOI:** 10.1186/s12957-016-0918-1

**Published:** 2016-06-17

**Authors:** Naoki Kaneko, Shintaro Kawano, Ryota Matsubara, Yuichi Goto, Teppei Jinno, Yasuyuki Maruse, Taiki Sakamoto, Yuma Hashiguchi, Masakazu Iida, Seiji Nakamura

**Affiliations:** Section of Oral and Maxillofacial Oncology, Division of Maxillofacial Diagnostic and Surgical Sciences, Faculty of Dental Science, Kyushu University, 3-1-1 Maidashi, Higashi-ku, Fukuoka 812-8582 Japan; Maxillofacial Diagnostic and Surgical Science, Department of Oral and Maxillofacial Rehabilitation, Course for Developmental Therapeutics, Kagoshima University Graduate School of Medical and Dental Sciences, 8-35-1 Sakuragaoka, Kagoshima, 890-8544 Japan

**Keywords:** Squamous cell carcinoma, Parathyroid hormone-related protein, Granulocyte colony-stimulating factor

## Abstract

**Background:**

Paraneoplastic syndrome generally results from tumor-derived hormones or peptides that cause metabolic derangements. Common metabolic conditions include hyponatremia, hypercalcemia, hypoglycemia, and Cushing’s syndrome. Herein, we report a very rare case of tongue carcinoma presenting with leukocytosis and hypercalcemia.

**Case presentation:**

A 57-year-old man was admitted to our hospital with tongue squamous cell carcinoma (cT4aN0M0, stage IV). He underwent radical resection following preoperative chemoradiotherapy, but locoregional recurrence was detected 2 months after surgery. He presented with marked leukocytosis and hypercalcemia with elevated serum levels of granulocyte colony-stimulating factor (G-CSF) and parathyroid hormone-related protein (PTHrP). He was therefore managed with intravenous fluids, furosemide, prednisolone, elcatonin, and pamidronate. However, the patient died 1 month later of carcinomatous pleuritis following distant metastasis to the lung. Immunohistochemical analyses of the resected specimens revealed positive staining for PTHrP and G-CSF in the cancer cells.

**Conclusions:**

In this case, it was considered that tumor-derived G-CSF and PTHrP caused leukocytosis and hypercalcemia.

## Background

Malignant tumors occasionally secrete hormonal factors that can cause some types of paraneoplastic symptoms [1–3]. Hypercalcemia is a relatively common paraneoplastic syndrome, and recent studies have demonstrated that parathyroid hormone-related protein (PTHrP) secreted by tumor cells can cause hypercalcemia in patients with malignant tumors [4, 5], while leukocytosis is a paraneoplastic syndrome caused by tumor-derived granulocyte colony-stimulating factor (G-CSF) [6]. However, the paraneoplastic production of both PTHrP and G-CSF by cancer cells is extremely rare. In this case report, we present a patient with tongue squamous cell carcinoma presenting with hypercalcemia and leukocytosis caused by tumor-derived PTHrP and G-CSF.

## Case presentation

**Fig. 1:**
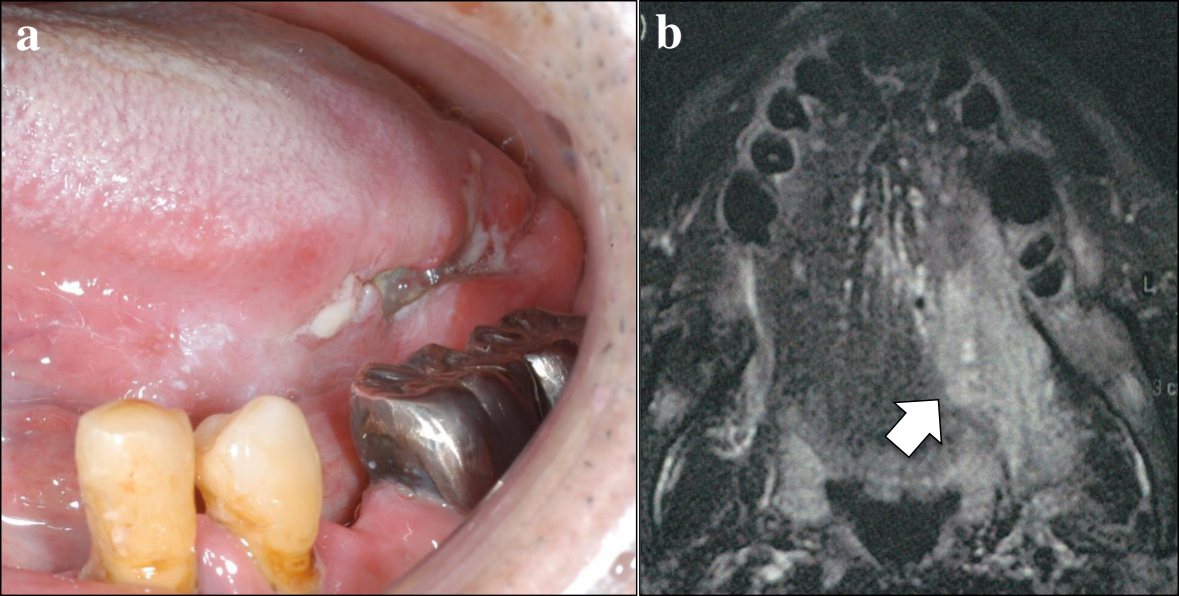
Intraoral and MRI findings. **a** Tumorous mass with ulcerative lesion on the left lateral border of the tongue. **b** Contrast-enhanced MRI findings. *White arrow* indicates tumorous mass

**Fig. 2:**
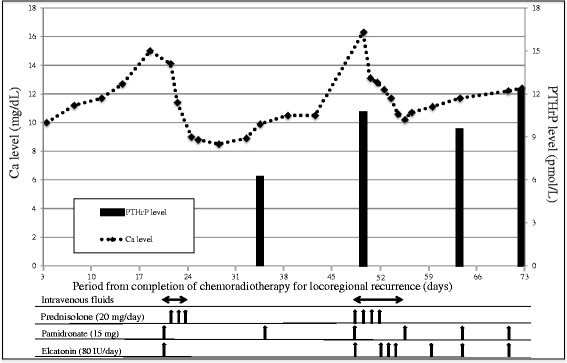
Time course of serum levels of calcium and PTHrP. Serum levels of calcium and PTHrP gradually increased in parallel after the completion of chemoradiotherapy for locoregional recurrence

**Fig. 3:**
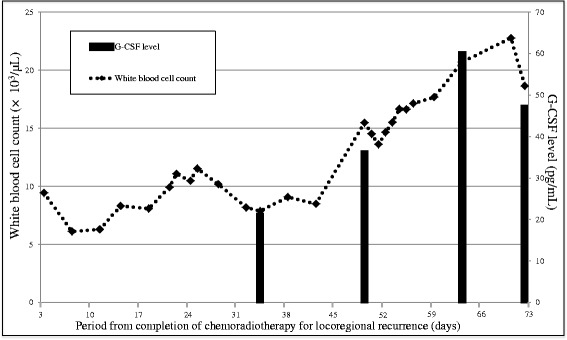
Time course of white blood cell count and serum G-CSF level. The number of white blood cells and G-CSF level gradually increased in parallel

**Fig. 4:**
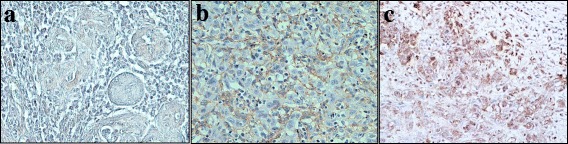
Immunohistochemical staining for PTHrP, G-CSF, and IL-6 in residual cancer cells. (×400). **a** PTHrP. **b** G-CSF. **c** IL-6

A 57-year-old man was referred to the Department of Oral and Maxillofacial Surgery, Kyushu University, with a chief complaint of a painful ulcerative lesion on the left lateral border of his tongue (Fig. 1a). Magnetic resonance imaging (MRI) revealed a tumorous mass occupying half of the tongue on the left side, extending to the lingual septum, and partially invading into the internal pterygoid muscle (Fig. 1b). No metastatic lymph nodes were found in the bilateral neck by palpation, ultrasonography, or computed tomography (CT). The patient had a medical history of diabetes mellitus. Pathological diagnosis of an incisional biopsy specimen indicated a moderately differentiated squamous cell carcinoma (SCC) (cT4aN0M0, stage IV). Planning CT was carried out followed by preoperative chemoradiotherapy including external beam irradiation to the primary tumor and neck in daily fractions of 2 Gy, five times weekly for 3 weeks, and oral administration of S-1 (120 mg/day) started 1 week prior to radiotherapy and continued throughout the radiotherapy period. One month after completing the preoperative chemoradiotherapy, the tumor was resected by subtotal glossectomy and segmental mandibulectomy under general anesthesia. Modified radical neck dissection and reconstruction using a rectus abdominis myocutaneous flap were performed simultaneously with tumor resection. Pathologically, four metastatic lymph nodes were identified: one in level I, one in level II, and two in level III. The histopathological response of the primary tumor to preoperative chemoradiotherapy was poor, and many residual carcinoma cells were noted in the muscular tissues in the resected specimens, though the surgical margin was tumor-free. Locoregional recurrence was detected by CT imaging 3 months after surgery, and the patient received further chemoradiotherapy (S-1, 120 mg/day; external beam irradiation in daily fractions of 2 Gy, five times weekly for 7 weeks). However, after completion of the chemoradiotherapy, the patient developed hypercalcemia (maximum serum calcium level, 16.3 mg/dL) and leukocytosis (maximum white blood cell count, 22.7 × 10^3^/μL). The serum levels of PTHrP and G-CSF increased in parallel with progression of his hypercalcemia and leukocytosis (Figs. 2 and 3). Immunohistochemical staining revealed expression of PTHrP and G-CSF in the residual cancer cells (Fig. 4a, b). Interleukin (IL)-6 expression was also detected in the cancer cells (Fig. 4c). Based on these clinical and pathological findings, the patient was diagnosed with hypercalcemia and leukocytosis associated with malignancy. He was subsequently managed with intravenous fluids, furosemide, prednisolone, elcatonin, and pamidronate. However, an F-18 fluorodeoxyglucose positron emission tomography (FDG-PET) scan disclosed multiple metastatic regions, including the pelvis, lung, femur, adrenal gland, sternal bone, and inguinal nodes, and he progressed to respiratory failure and died of carcinomatous pleuritis 1 month later.

## Conclusions

We present a very rare case of a patient with tongue squamous cell carcinoma presenting with hypercalcemia and leukocytosis. The serum levels of PTHrP and G-CFS were markedly elevated in the patient, in line with progression of his hypercalcemia and leukocytosis. Furthermore, immunohistological staining revealed PTHrP and G-CSF expression in the residual carcinoma cells. These findings suggested that hypercalcemia and leukocytosis in this patient were associated with tumor-derived PTHrP and G-CSF.

Yoneda et al. reported only five cases (2.2 %) presenting with both hypercalcemia and leukocytosis among 225 oral malignancies [7], though they did not demonstrate if the hypercalcemia and leukocytosis were caused by tumor-derived PTHrP and G-CSF. To the best of our knowledge, there have been only 13 recorded cases of head and neck tumors that produced both PTHrP and G-CSF, including the current case (Table 1) [8–16], in all of which patients presented with both hypercalcemia and leukocytosis. Pathologically, squamous cell carcinoma was the most frequent type of tumor (10 cases, 76.9 %), but only three cases of tongue squamous cell carcinoma have been reported [11, 12]. Furthermore, except for a patient with a benign ameloblastoma, 11 of the 12 previous cases had a poor prognosis. In the patient with hypopharyngeal carcinoma, the serum levels of both PTHrP and G-CSF were remarkably decreased after radical resection and the patient was rescued [16]. The present and previous findings thus suggest that increased serum levels of both PTHrP and G-CSF are indicative of a poor prognosis in patients with head and neck tumors.

**Table 1:** Case reports of head and neck tumors producing both PTHrP and G-CSF

No. [ref]	Sex	Age (year)	Primary site	Histological type	TNM classification	Differentiation	Serum Ca	Leukocyte (/μL)	Serum PTHrP (pmol/L) (normal range)	Serum G-CSF (pg/mL) (normal range)	Main metastases	Outcome
1 [8]	Female	65	Thyroid gland	Anaplastic carcinoma	–	–	13.8	142,000	4.02 (<1.1)	318 (<30)	Cervix, lung, liver	death
2 [9]	Male	48	Tonsil	SCC	–	–	11.4	62,100	533.3 (<55.3)	3450 (<30)	Cervix, lung, liver,	Death
3 [10]	Female	63	Thyroid gland	Follicular and papillary carcinoma	T2N1bM1	Well	13.4	34,700	3.9	196	Lung, bone	Death
4 [11]	Male	73	Buccal mucosa	SCC	T4N3M1	Poor	15.1	40,200	108.5 (<55.3)	1800 (<30)	Lung	Death
5 [11]	Male	60	Gingiva	SCC	T4N2M0	Well	18.1	15,800	227.9 (<55.3)	34.3 (<30)	Bone, lung	Death
6 [11]	Male	82	Tongue	SCC	T3N2M0	Poor	14.7	79,000	132 (<55.3)	274 (<30)	Lung	Death
7 [11]	Male	65	Buccal mucosa	SCC	T4N2M0	Poor	16.2	34,600	255 (<55.3)	211 (<30)	Lung, liver, pancreas	Death
8 [12]	Male	61	Tongue	SCC	T1N0MX	Well	28.5	31,300	3109 (<16) [pg/mL]	285 (<20) (pleural fluid)	Cervix, lung	Death
9 [13]	Male	65	Larynx	SCC	T4N3M0	–	18	60,000	5.6 (<0.5)	222 (<38)	Cervix, lung	Death
10 [14]	Female	57	Hypopharynx	SCC	T3N2bMX	Poor	14.2	46,300	12.4 (<1.1)	111 (<39)	Cervix, lung, liver, bone, kidney, skin	Death
11 [15]	Female	32	Mandible	Ameloblastoma	–	–	11.3	37,200	14.7 (<1.1)	68 (<30)	–	Survive
12 [16]	Male	76	Hypopharynx	SCC	T4aN2cM0	Moderate	11.2	20,230	3.1 (<1.1)	213 (<39)	Cervix, esophagus	Survive
Present case	Male	57	Tongue	SCC	T4aN0M0	Moderate	16.3	22,770	12.2 (<1.1)	60 (<39)	Cervix, lung, bone, femur, adrenal gland, groin	Death

Hypercalcemia can be categorized as humoral hypercalcemia of malignancy (HHM) or local osteolytic hypercalcemia (LOH) [17]. HHM is caused by tumor-derived PTHrP, while LOH is mainly caused by osteolysis associated with bone metastasis. The present patient demonstrated multiple bone metastases on FDG-PET with elevated serum PTHrP levels, suggesting the existence of both HHM and LOH in this case. The clinical manifestations of hypercalcemia affect the neuromuscular, renal, gastrointestinal, skeletal, and cardiovascular systems [18], and anti-hypercalcemic therapy should therefore be initiated as soon as possible to avoid hypercalcemic crisis [19]. In this patient, saline infusion (2000–3000 mL/day) with concomitant loop diuretics (furosemide) was administered to increase calcium excretion and reduce serum levels as rapidly as possible. We also administered calcitonin (a naturally occurring peptide hormone that inhibits bone resorption and increases renal calcium excretion) and a bisphosphonate (alendronate) to treat the hypercalcemia [20]. Bisphosphonates are effective therapeutic agents for hypercalcemia that inhibit osteoclast differentiation and function [21]. We also used prednisolone to reduce gastrointestinal calcium absorption [22]. However, although the patient’s calcium levels were normalized by this therapy, subsequent hypercalcemic exacerbation developed. Yamazaki et al. demonstrated an average survival period of 39.0 days from the diagnosis of hypercalcemia in patients with oral cancer treated with bisphosphonates or calcitonin, compared with 19.5 days in untreated patients [11]. These results suggest that the prognosis of patients with tumor-derived PTHrP and G-CSF is extremely unfavorable, though anti-hypercalcemic therapy may slightly improve life expectancy.

Numerous previous studies have shown that G-CSF is responsible for paraneoplastic leukocytosis [6, 7, 23–25]. However, the mechanisms responsible for the production of these hormonal factors by tumor cells remain unknown. Recent studies have suggested that some inflammatory cytokines, including IL-6, may be associated with G-CSF production by malignant cells [26]. Moreover, hypercalcemia has been associated with cosecretion of PTHrP and IL-6 [27]. We previously showed that increased IL-6 expression in cancer cells predicted a poor response to chemoradiotherapy and an unfavorable prognosis in patients with oral SCC [28]. The current patient demonstrated a poor histological response to preoperative chemoradiotherapy and strong IL-6 expression in the residual cancer cells. These results suggested that IL-6 may play a key role in resistance to chemoradiotherapy and production of PTHrP and G-CSF in the cancer cells. However, further in vitro and in vivo studies are needed to clarify the association between IL-6 expression in oral squamous cell carcinoma and the production of PTHrP and G-CSF.

## Abbreviations

FDG-PET, F-18 fluorodeoxyglucose positron emission tomography; G-CSF, granulocyte colony-stimulating factor; HHM, humoral hypercalcemia of malignancy; IL-6, interleukin 6; LOH, local osteolytic hypercalcemia; PTHrP, parathyroid hormone-related protein; SCC, squamous cell carcinoma

## References

[1] Robinson WA (1974). Granulocytosis in neoplasia. Ann NY Acad Sci.

[2] Broadus AE, Mangin M, Ikeda K, Insogna KL, Weir EC, Burtis WJ (1988). Humoral hypercalcemia of cancer. Identification of a novel parathyroid hormone-like peptide. N Engl J Med.

[3] Isidori AM, Kaltsas GA, Pozza C, Frajese V, Newell-Price J, Reznek RH (2006). The ectopic adrenocorticotropin syndrome: clinical features, diagnosis, management, and long-term follow-up. J Clin Endocrinol Metab.

[4] Suva LJ, Winslow GA, Wettenhall RE, Hammonds RG, Moseley JM, Diefenbach-Jagger H (1987). A parathyroid hormone-related protein implicated in malignant hypercalcemia: cloning and expression. Science.

[5] Mangin M, Ikeda K, Dreyer BE, Broadus AE (1989). Isolation and characterization of the human parathyroid hormone-like peptide gene. Proc Natl Acad Sci U S A.

[6] Asano S, Urabe A, Okabe T, Sato N, Kondo Y (1977). Demonstration of granulopoietic factor(s) in the plasma of nude mice transplanted with a human lung cancer and in the tumor tissue. Blood.

[7] Yoneda T, Nishimura R, Kato I, Masatoshi O, Masaaki T, Masayoshi S (1991). Frequency of the hypercalcemia-leukocytosis syndrome in oral malignancy. Cancer.

[8] Yazawa S, Toshimori H, Nakatsuru K, Katakami H, Takemura J, Matsukura S (1995). Thyroid anaplastic carcinoma producing granulocyte-colony-stimulating factor and parathyroid hormone-related protein. Intern Med.

[9] Murao T, Takaba S, Fujita T (1997). A case of squamous cell carcinoma of the tonsil with high concentration of serum granulocyte colony stimulating factor and parathyroid hormone-related protein. J Okayama Surg Pathol Assoc.

[10] Kunisue H, Tanaka K, Sonoo H, Kurebayashi J, Shimozuma K, Mikami Y (2000). Transformation to undifferentiated from differentiated thyroid cancer associated with remarkable leukocytosis and hypercalcemia: report of a case. J Japanese Surg Assoc.

[11] Yamazaki H, Ota Y, Karakida K, Sekiya R, Mizusawa N, GOTO J (2000). Hypercalcemia in patients with oral cancer. Japan Soc Head Neck Cancer.

[12] Obara S, Yoshimura Y (2001). A case of tongue carcinoma showing leukocytosis and hypercalcemia with the production of G-CSF and PTHrP. Jpn J Oral Maxillofac Surg.

[13] Tanaka K, Nibu K (2005). Laryngeal squamous cell carcinoma with ectopic production of granulocyte colony-stimulating factor and parathyroid hormone-related protein. Int J Clin Oncol.

[14] Tamura K, Yoshinaga T, Tanino M, Kimura T, Yamada N, Nishimura M (2008). Hypopharyngeal squamous cell carcinoma producing both granulocyte colony-stimulating factor and parathyroid hormone-related protein. Pathol Int.

[15] Ota Y, Aoki T, Otsuru M, Hirabayashi K, Nakamura N, Tsukinoki K (2011). Huge ameloblastoma associated with hypercalcemia, leukocytosis, and elevated tumor markers via production of parathyroid hormone-related protein and granulocyte colony-stimulating factor. J Oral Maxillofac Surg.

[16] Matsuo M, Rikimaru F, Higaki Y, Masuda M (2013). A case of G-CSF producing hypopharyngeal carcinoma. Japanese J Head Neck Cancer.

[17] Rosol TJ, Capen CC (1992). Biology of disease: mechanisms of cancer-induced hypercalcemia. Lab Invest.

[18] Bajorunas DR (1990). Clinical manifestations of cancer-related hypercalcemia. Semin Oncol.

[19] Iwase M, Takemi T, Manabe M, Nagumo M (2003). Hypercalcemic complication in patients with oral squamous cell carcinoma. Int J Oral Maxillofac Surg.

[20] Hosking DJ, Gilson D (1984). Comparison of the renal and skeletal actions of calcitonin in the treatment of severe hypercalcemia of malignancy. Q J Med.

[21] Watters J, Gerrard G, Dodwell D (1996). The management of malignant hypercalcemia. Drugs.

[22] Pelosof LC, Gerber DE (2010). Paraneoplastic syndromes: an approach to diagnosis and treatment. Mayo Clin Proc.

[23] Horii A, Shimamura K, Honjo Y, Mitani K, Miki T, Takashima S (1997). Granulocyte colony stimulating factor-producing tongue carcinoma. Head Neck.

[24] Ito T, Shimamura K, Shoji K, Akatsuka A, Kiryu Y, Tamaoki N (1993). Urinary bladder carcinoma producing granulocyte colony stimulating factor (G-CSF): a case report with immunohistochemistry. Virchows Archiv A Pathol Anat.

[25] Ota S, Kato A, Kobayashi H, Yonezumi M, Yamaguchi J, Musashi M (1998). Monoclonal origin of an esophageal carcinosarcoma producing granulocyte-colony stimulating factor. Cancer.

[26] Inoue M, Minami M, Fujii Y, Matsuda H, Shirakura R, Kido T (1997). Granulocyte colony-stimulating factor and interleukin-6-producing lung cancer cell line. LCAM J Surg Oncol.

[27] Asanuma N, Hagiwara K, Matsumoto I, Matsuda M, Nakamura F, Kouhara H (2002). PTHrP-producing tumor: squamous cell carcinoma of the liver accompanied by humoral hypercalcemia of malignancy, increased IL-6 and leukocytosis. Intern Med.

[28] Jinno T, Kawano S, Maruse Y, Matsubara R, Goto Y, Sakamoto T (2015). Increased expression of interleukin-6 predicts poor response to chemoradiotherapy and unfavorable prognosis in oral squamous cell carcinoma. Oncol Rep.

