# Erratum to: Tongue squamous cell carcinoma producing both parathyroid hormone-related protein and granulocyte colony-stimulating factor: a case report and literature review

**DOI:** 10.1186/s12957-016-0945-y

**Published:** 2016-07-19

**Authors:** Naoki Kaneko, Shintaro Kawano, Ryota Matsubara, Yuichi Goto, Teppei Jinno, Yasuyuki Maruse, Taiki Sakamoto, Yuma Hashiguchi, Masakazu Iida, Seiji Nakamura

**Affiliations:** Section of Oral and Maxillofacial Oncology, Division of Maxillofacial Diagnostic and Surgical Sciences, Faculty of Dental Science, Kyushu University, 3-1-1 Maidashi, Higashi-ku, Fukuoka 812-8582 Japan; Maxillofacial Diagnostic and Surgical Science, Department of Oral and Maxillofacial Rehabilitation, Course for Developmental Therapeutics, Kagoshima University Graduate School of Medical and Dental Sciences, 8-35-1 Sakuragaoka, Kagoshima, 890-8544 Japan

## Erratum

After publication of the original article [1] it was brought to our attention that the funding information was not correctly reflected in the article.

The original Funding information contains an incorrect number. The original sentence reads as:

This work was supported by a Grant-in-Aid (26463014, 60615798, and 26670869) from the Japanese Ministry of Education, Culture, Sports, Science and Technology.

The revised sentence, with the correct number reads as follows:

This work was supported by a Grant-in-Aid (26463014, 26861729, and 26670869) from the Japanese Ministry of Education, Culture, Sports, Science and Technology.

Please note that the number 60615798 should instead be **26861729**.
